# The spatial distribution of health vulnerability to heat waves in Guangdong Province, China

**DOI:** 10.3402/gha.v7.25051

**Published:** 2014-10-21

**Authors:** Qi Zhu, Tao Liu, Hualiang Lin, Jianpeng Xiao, Yuan Luo, Weilin Zeng, Siqing Zeng, Yao Wei, Cordia Chu, Scott Baum, Yaodong Du, Wenjun Ma

**Affiliations:** 1 Guangdong Provincial Center for Disease Control and Prevention, Guangzhou, China; 2 Guangdong Provincial Institute of Public Health, Guangdong Provincial Center for Disease Control and Prevention, Guangzhou, China; 3 Environment and Health, Guangdong Provincial Key Medical Discipline of Twelfth Five-Year Plan, Guangzhou, China; 4 Griffith School of Environment, Griffith University, Brisbane, Australia; 5 Guangdong Provincial Climate Center, Guangzhou, China

**Keywords:** vulnerability assessment, heat waves, climate change, analytic hierarchy process, principal component analysis

## Abstract

**Background:**

International literature has illustrated that the health impacts of heat waves vary according to differences in the spatial variability of high temperatures and the social and economic characteristics of populations and communities. However, to date there have been few studies that quantitatively assess the health vulnerability to heat waves in China.

**Objectives:**

To assess the spatial distribution of health vulnerability to heat waves in Guangdong Province, China.

**Methods:**

A vulnerability framework including dimensions of exposure, sensitivity, and adaptive capacity was employed. The last two dimensions were called social vulnerability. An indicator pool was proposed with reference to relevant literatures, local context provided by relevant local stakeholder experts, and data availability. An analytic hierarchy process (AHP) and a principal component analysis were used to determine the weight of indicators. A multiplicative vulnerability index (VI) was constructed for each district/county of Guangdong province, China.

**Results:**

A total of 13 items (two for exposure, six for sensitivity, and five for adaptive capacity) were proposed to assess vulnerability. The results of an AHP revealed that the average VI in Guangdong Province was 0.26 with the highest in the Lianzhou and Liannan counties of Qingyuan (VI=0.50) and the lowest in the Yantian district of Shenzhen (VI=0.08). Vulnerability was gradiently distributed with higher levels in northern inland regions and lower levels in southern coastal regions. In the principal component analysis, three components were isolated from the 11 social vulnerability indicators. The estimated vulnerability had a similar distribution pattern with that estimated by AHP (Intraclass correlation coefficient (ICC)=0.98, *p*<0.01).

**Conclusions:**

Health vulnerability to heat waves in Guangdong Province had a distinct spatial distribution, with higher levels in northern inland regions than that in the southern coastal regions.

The fifth assessment report of the Intergovernmental Panel on Climate Change (IPCC) has argued that, as a result of global warming, many regions worldwide are expected to witness increasing numbers of extreme temperature events or heat wave periods (1). Within the Chinese context, a heat wave is defined by the Meteorological Administration of China as a period of daily maximum temperature exceeding 35°C and lasting three consecutive days or more. For Southern China, which includes the Guangdong Province, historical meteorological data reveal that since the mid-1990s, heat wave events have become more and more frequent (2). Guangdong Province has a subtropical climate, where the average temperature is higher than most parts of mainland China. In the past 60 years, Guangdong Province has experienced significant changes to its weather patterns. The average increase in heat wave days (daily maximum temperature≥35°C) was 0.27 days/year. The average number of annual heat wave days during the 1970s was less than 10 days; however, this has increased rapidly to more than 20 days per year after 1998 (3).

The impact of heat waves on human health including increases in mortality and morbidity has been widely noted (4–8). For example, heat waves that impacted on France during 2003 resulted in deaths in excess of 15,000 (9). In addition, the negative impact caused by heat waves might last for several days or longer (10–12). While aggregate level data provide useful baseline information, public health responses to heat wave events require more nuanced approaches and information in order to develop useful policy and programs that address the vulnerability of local populations and communities. In this sense, and following the existing international literature, vulnerability is seen to be a function of the level of exposure to a heat wave event (EI), the level of sensitivity to a heat wave event (SI), and the level of adaptive capacity (AI) of populations (13, 14). Exposure is defined as the nature and degree to which a system is exposed to significant climatic variations, while sensitivity is the degree to which a system is affected, either positively or negatively by a climate event. The combination of exposure and sensitivity provides a measure of potential impact or gross vulnerability. The third component, adaptive capacity, is the ability of a system to adjust to a climate event, to moderate potential damages, to take advantage of opportunities, or to cope with the consequence. Adaptive capacity mediates potential impact or gross vulnerability to provide an indication of net vulnerability. Exposure is seen as a function of climate, while sensitivity and adaptive capacity are related to the socio-economic characteristics of a given population. In this way, sensitivity and adaptive capacity can also be referred to as social vulnerability and the combination of the two represents a social vulnerability index (SVI) (13). Translating this into an understanding of how vulnerability to heat waves impact differently upon regions of communities requires a focus on the spatially heterogeneous nature of vulnerability and its separate components.

Within the international literature, there have been many studies that have evaluated the regional or spatial variation in vulnerability to heat waves (15–18). In contrast to these studies, the research literature in China has been largely devoid of regional or spatial assessments of heat wave vulnerability. For Guangdong Province, like other regions, climatic variation and unbalanced socio-economic development at a disaggregated spatial or regional level is likely to result in significant variation in heat wave vulnerability. This paper addresses this issue and contributes to an understanding of the potential spatial or regional variation in heat wave vulnerability at district/county level for Guangdong Province, China. It does so by considering the components of vulnerability discussed above and using spatial data on climatic and socio-economic characteristics develops a regional level index for the province.

## Methods

### Vulnerability framework

**Fig. 1:**
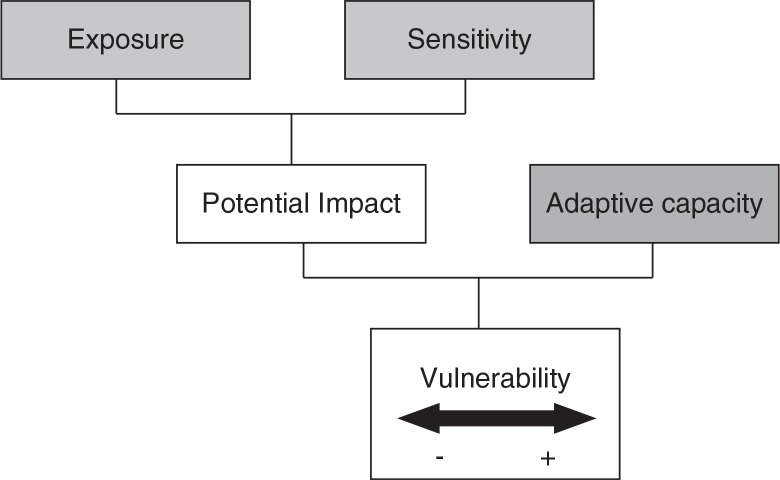
Framework of vulnerability.

The literature on climate change vulnerability recognizes a wide range of potential measures and methods (19). We employed a framework recognized by the IPCC (Fig. 1) (13), in which vulnerability was a function of exposure, sensitivity, and adaptive capacity (Equation 1), and developed indicators of all three dimensions. A multiplicative model was used to develop vulnerability index (VI) for each district/county (20).

1 $$\text{V}{\text{I}}_{j}=\text{E}{\text{I}}_{j}*\frac{\left(1+\text{S}{\text{I}}_{j}+\text{A}{\text{I}}_{j}\right)}{\text{n}}$$

where VI_*j*_ indicates the overall VI to heat waves in district/county *j*. It is estimated mathematically combining the following components: EI_*j*_ is the component measuring the level of exposure to heat waves within district/county *j*; SI_*j*_ is the sensitivity index for district/county *j*; AI_*j*_ is the adaptive capacity index for district/county *j*; and *n* is the total number of components included in the sensitivity index and adaptive capacity index.

Equation 1 can be interpreted as follows. Vulnerability in terms of heat waves depends on the spatial variation of exposure (a necessary condition) plus the spatially differentiated socio-economic characteristics of the population. Local heat wave exposure is considered to be a necessary condition for vulnerability. Once this condition is satisfied, the overlaps with the range of components that make up sensitivity and adaptive capacity define specific situations of heat wave vulnerability. The combined variation in exposure, sensitivity, and adaptive capacity therefore result in a spatially differentiated understanding of vulnerability across Guangdong Province.

### Indicator selection for each dimension

An indicator pool was generated with reference to a range of existing studies (17, 21–25) and consultations with expert stakeholders. First, we searched related literature databases, including MEDLINE, PubMed, and China National Knowledge Infrastructure (CNKI). All studies that used the similar methodology and vulnerability framework were included. Second, all related indicators were independently selected by two authors, and minor discrepancies were resolved by discussion. Meanwhile, stakeholder expert consultations were also conducted to collect vulnerability indicators. These experts were selected from public health, meteorology, and social sciences fields. Finally, all collected indicators were gathered to generate a primary indicator pool which included 46 indicators (8 for exposure, 21 for sensitivity, and 17 for adaptive capacity). Nine experts from the fields of public health, meteorology, and social sciences were invited to select appropriate indicators for each dimension from the indicator pool based on the following three principles: 1) indicators should sensitively reflect the vulnerability of a region or population to heat waves; 2) indicators should be easily implemented in practical work and have no limits imposed by data availability; 3) indicators should reflect being used in existing studies of other countries and regions.

Three meteorology experts mainly selected indicators that could explain the heat-related exposure. Three public health experts mainly selected indicators which could explain the heat-related sensitivity. Three social science experts mainly selected indicators which should reflect the social vulnerability to heat waves. After preliminary selection of all indicators, experts discussed the collective suite of indicators, deleted indicators with poor representation or high correlations, and improved indicators that required some modification to make them appropriate for this study.

### Data collection

Sensitivity and adaptive capacity indicators were obtained from the National Sixth Census (26), Guangdong Statistical Yearbook (27), and Health Statistics Year book of Guangdong Province (28). Exposure indicators were obtained from Guangdong Meteorological Bureau.

### Standardization and weight determination of each indicator

The final index was developed with reference to Equation 1. Prior to the index calculation, all individual indicators were standardized to remove potential issues associated with using indicators measured at different scales. Standardization was undertaken with reference to the following formula:

2 $$\text{Std}(I_{ij})=\frac{I_{ij}}{\max_{i}}$$

in which, Std(*I*_*ij*_) is the standardized indicator *i* for district/county *j*, *I*_*ij*_ is the unstandardized indicator *i* for district/county *j*, and max_*i*_ is the maximum value of indicator *i* among all districts/counties.

Using this standardization approach, each individual indicator was rescaled into a common measurement scale that ranged between 0 and 1.

Before calculating the standardized score of each dimension, a subjective (AHP) and an objective method (principal component analysis method) were employed to determine the weight of each indicator.

#### Analytic hierarchy process

Nine stakeholder experts from public health, meteorology, or social science fields were invited to determine the relative importance of all indicators in each dimension. An AHP method was then used to generate weight for each indicator based on the relative importance in each dimension of the VI (29). An expert could subjectively judge the relative importance between indicators following a 1–9 fundamental scale, in which ‘1’ indicated equal importance, and ‘9’ indicated extreme importance. A judgment matrix would be obtained for each dimension from each expert. A consistency test using a consistence ratio (CR) was used to test whether the sorting results were logically consistent. The scoring results could be considered as consistent when CR<0.10. The final weight for each indicator was an average of the results given by all experts.

#### Principal component analysis

The indicators for the dimensions of sensitivity and adaptive capacity are demographic or socio-economic factors and represented the overall SVI. As these individual dimensions are likely to be inter-related, a principal component analysis was used to isolate these inter-related indicators and determine their weights (30). All indicators entered into a correlation matrix, and a varimax or thogonal rotation with Kaiser normalization was applied. The criterion for the retention of a component was an Eigen value greater than one. The weight of each indicator was calculated based on their factor scores and the proportion of variance of each component. As there were only two indicators for the exposure dimension, their weights were unlikely to be determined by principal component analysis, and were determined by AHP only.

### Calculation of VI

In order to apply the weighted indicators to the formula in Equation 1, separate indicators for EI, SI, and AI were calculated using the following formulas:

3 $$\text{EI}=w_{E_{1}}\cdot E_{1}+w_{E_{2}}\cdot E_{2}+\ldots +w_{E_{n}}\cdot E_{n}$$

4 $$\text{SI}=w_{S_{1}}\cdot S_{1}+w_{S_{2}}\cdot S_{2}+\ldots +w_{S_{n}}\cdot S_{n}$$

5 $$\text{AI}=w_{A_{1}}\cdot A_{1}+w_{A_{2}}\cdot A_{2}+\ldots +w_{A_{n}}\cdot A_{n}$$

where *E*_*1*_ *–E*_*n*_ were indicators 1~n of exposure, *S*_1_ *–S*_*n*_ were indicators 1~n of sensitivity, *A*_1_ *–A*_n_ were indicators 1~n of adaptive capacity, and *w* was the weight of each indicator. In the AHP, VI of each district/county was estimated based on Equation (1). In the principal component analysis, a transformed equation (Equation 6) was employed to estimate each district/county's VI, in which SVI was the sum of SI and AI, and C1_*j*_, C2_*j*_ and C3_*j*_ were the three component scores in district/county *j*.

6 $$\text{V}{\text{I}}_{j}=\text{E}{\text{I}}_{j}*\frac{\left(1+\text{S}{\text{I}}_{j}+A{\text{I}}_{j}\right)}{n}=\text{E}{\text{I}}_{j}*\frac{\left(1+\text{SV}{\text{I}}_{j}\right)}{n}=\text{E}{\text{I}}_{j}*\frac{\left(1+\text{C}1_{j}+\text{C}2_{j}+\text{C}3_{j}\right)}{3}$$

**Appendix Fig. 1:**
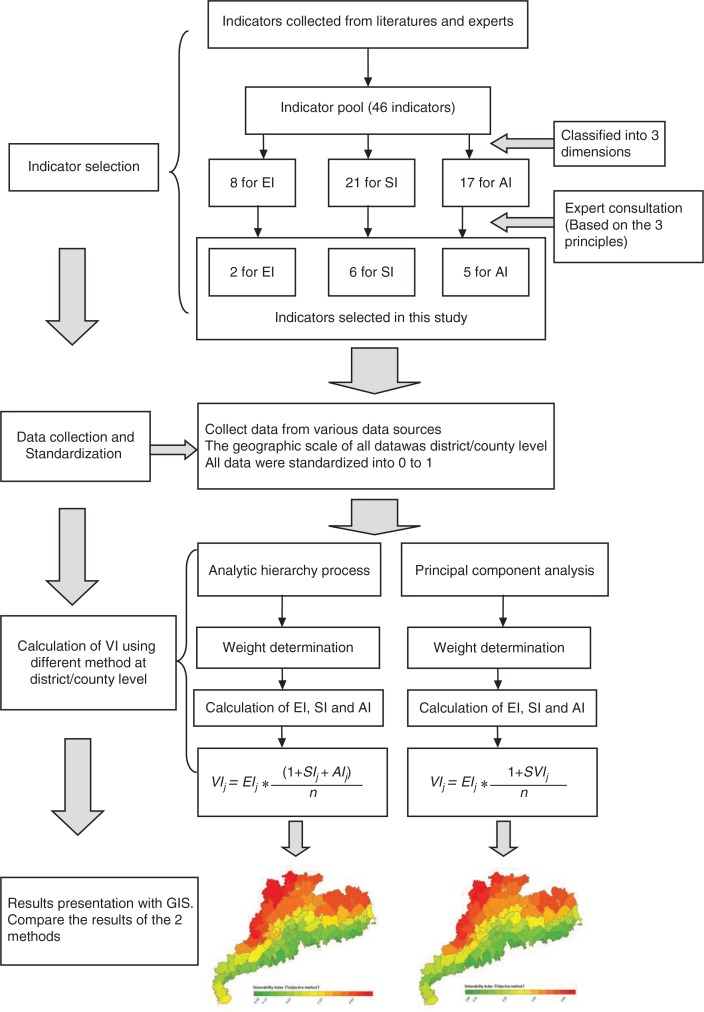
The flow diagram of the assessment process.

The higher the VI value, the greater the adverse impact caused by the heat waves in this district/county. A geographic information system (ArcGIS) was used to display the distribution of VIs among 124 districts/counties of Guangdong Province. The whole process of assessment could be seen in Appendix Fig. 1.

### Sensitivity analysis

Intraclass correlation coefficient (ICC) was used to assess the consistence of VIs of 124 districts/counties calculated by the subjective and objective methods.

## Results

A total of 13 indicators (six for sensitivity, five for adaptive capacity, and two for exposure) were finalized in this study to assess the vulnerability to heat waves at a district/county level in Guangdong Province. Table 1 shows the general characteristics of each indicator.

**Table 1:** Characteristics of selected indicators in each dimension

			Value		
	
Indicators	Data source	Time of data collection	Min	Median	Max
Sensitivity Index (SI)					
S1: % of population older than 65 years	National sixth Census	2010	1.0	7.3	12.2
S2: % of population less than 5 years	National sixth Census	2010	0.8	6.7	12.3
S3: % of immigrant population	National sixth Census	2010	0.9	9.4	95.3
S4: Unemployment rate (%)	National sixth Census	2010	0.2	0.5	1.0
S5: % of population engaged in agriculture	National sixth Census	2010	0.1	57.7	86.3
S6: Infant mortality rate (%)	National sixth Census	2010	0.2	1.1	7.8
Adaptive capacity Index (AI)					
A1: % of people who are health professionals	Guangdong Statistical Yearbook	2012	0.1	0.3	2.9
A2: GDP per capita (Yuan)	Guangdong Statistical Yearbook	2012	5,788	24,372	755,620
A3: % of households with per capita living area less than 8 m^2^	National sixth Census	2010	57.1	86.5	97.5
A4: % of harmless sanitary latrines	Health Statistics Year Book of Guangdong Province	2011	57.9	74.2	100.0
A5: % of illiterate in the population older than 15 years old	National sixth Census	2010	0.9	6.6	16.9
Exposure Index (EI)					
E1: Annual average temperature growth	Guangdong Meteorological Bureau	1975 to 2010	1.2	3.4	5.3
E2: Number of days with the daily maximum temperature over 35°C (day per year)	Guangdong Meteorological Bureau	1975 to 2010	3.7	12.4	28.4

### Results of AHP method

**Fig. 2:**
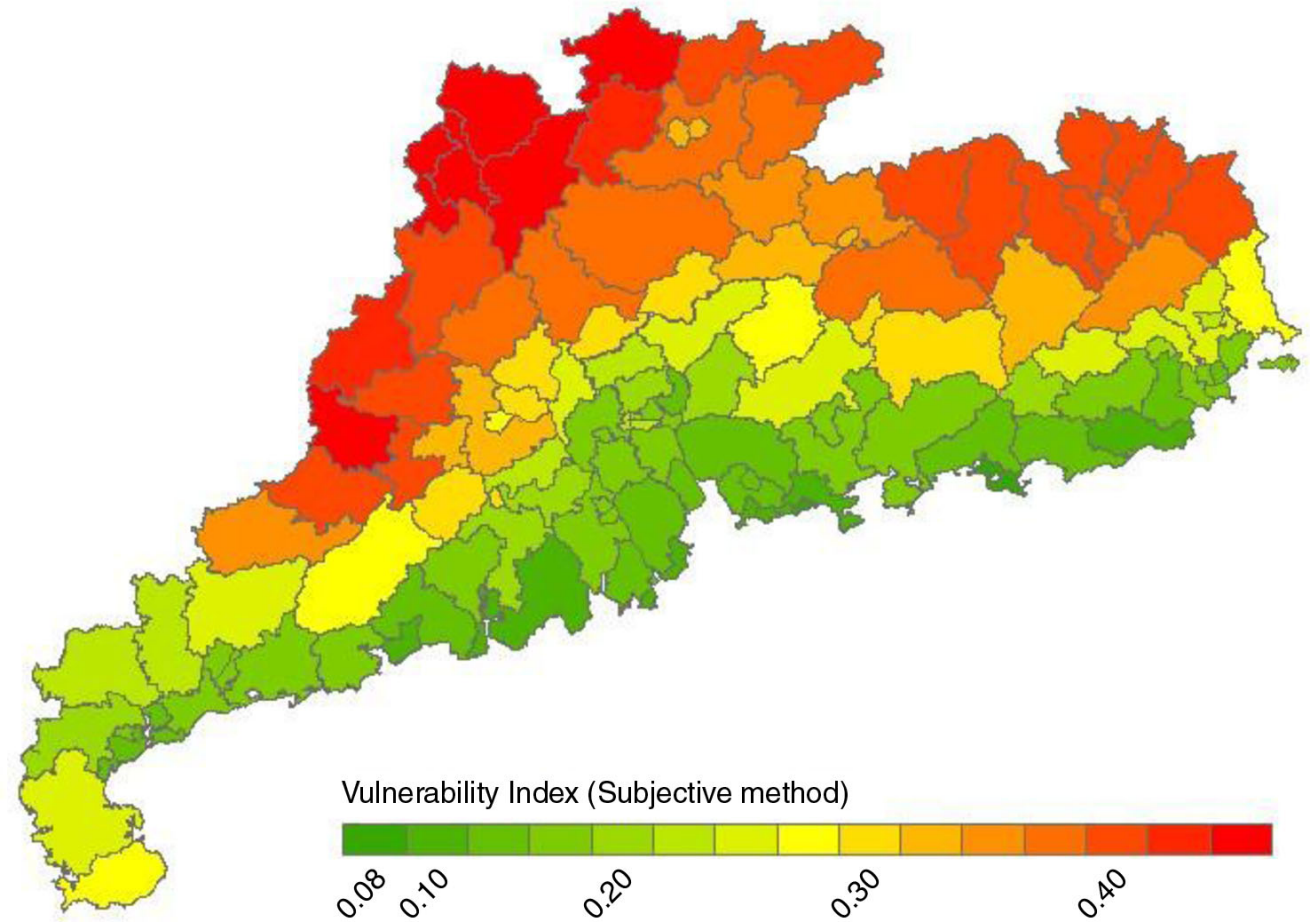
The distribution of vulnerability to heat waves among 124 counties/districts in Guangdong Province (analytic hierarchy process method).

Weight for each indicator was given individually by the nine expert stakeholders and then the average weight was calculated (Table 2). The result was consistent with all CRs<0.10. Figure 2 shows the distribution of 124 districts/counties’ VIs in Guangdong Province. The average score of VI was 0.26 with the highest in Lianzhou and Liannan counties of Qingyuan (VI=0.50) and the lowest in Yantian district of Shenzhen (VI=0.08). The VIs were gradiently distributed with higher in northern inland regions and lower in southern coast regions (Fig. 2).

**Table 2:** Weight of each indicator determined by expert scoring and analytic hierarchy process

	Experts									
		
Indicators	A	B	C	D	E	F	G	H	I	Average weight
Sensitivity Index (SI)										
S1: % of population older than 65 years	0.32	0.14	0.17	0.15	0.33	0.48	0.35	0.34	0.46	0.305
S2: % of population less than 5 years	0.17	0.05	0.08	0.07	0.08	0.10	0.34	0.34	0.22	0.160
S3: % of immigrant population	0.05	0.31	0.34	0.03	0.04	0.06	0.06	0.02	0.10	0.113
S4: Unemployment rate (%)	0.09	0.13	0.27	0.03	0.04	0.08	0.10	0.04	0.10	0.097
S5: % of population engaged in agricultural	0.05	0.33	0.08	0.27	0.19	0.11	0.09	0.08	0.06	0.141
S6: Infant mortality rate (%)	0.32	0.05	0.06	0.45	0.33	0.18	0.06	0.16	0.05	0.184
*CR* value of consistence test	0.08	0.09	0.09	0.09	0.02	0.10	0.10	0.04	0.05	
Adaptive capacity Index (AI)										
A1: % of people who are health professionals	0.04	0.26	0.25	0.24	0.11	0.32	0.07	0.21	0.06	0.174
A2: GDP per capita	0.33	0.35	0.47	0.48	0.05	0.28	0.17	0.45	0.44	0.334
A3: % of households with per-capita living area less than 8 m^2^	0.13	0.29	0.09	0.08	0.19	0.05	0.58	0.09	0.33	0.203
A4: % of harmless sanitary latrines	0.08	0.05	0.11	0.03	0.11	0.28	0.14	0.03	0.08	0.101
A5: % of illiterate in the population older than 15 years old	0.42	0.06	0.08	0.17	0.55	0.07	0.05	0.21	0.09	0.188
*CR* value of consistence test	0.07	0.09	0.09	0.09	0.09	0.01	0.10	0.05	0.06	
Exposure Index (EI)										
E1: Annual average temperature growth	0.17	0.14	0.20	0.20	0.17	0.13	0.13	0.75	0.17	0.228
E2: Number of days with the daily maximum temperature over 35°C	0.83	0.86	0.80	0.80	0.83	0.87	0.87	0.25	0.83	0.772
*CR* value of consistence test	0.00	0.00	0.00	0.00	0.00	0.00	0.00	0.00	0.00	

### Results of principal component analysis method

Among the 11 social vulnerability indicators, three components were isolated by a principal component analysis. The three components were accounted for 73.2% of the total variance, with component 1, 2, and 3, respectively, explaining 41.5, 18.3, and 13.3% of the total variance. The corresponding numbers of indicators for these three components were 7, 3, and 1. Each indicator's weight was calculated according to the results of principal component analysis (Table 3).

**Table 3:** The weight of each indicator in dimension sensitivity and adaptive capacity

	Rotated component matrix			
		
Indicator	C1	C2	C3	Weight
% of immigrant population	0.903	0.169	0.238	0.31
% of population engaged in agricultural	0.885	0.282	−0.125	0.27
% of population less than 5 years of age	0.879	−0.144	0.160	0.23
% of illiterate in the population older than 15 years old	0.781	0.323	0.116	0.28
% of people who are health professionals	0.750	−0.113	−0.346	0.13
% of population older than 65 years of age	0.563	0.441	0.510	0.30
GDP per capita	0.494	0.194	0.277	0.21
% of households with per-capita living area less than 8 m^2^	−0.192	0.822	−0.044	0.09
Infant mortality rate (%)	0.300	0.694	0.007	0.20
% of harmless sanitary latrines	0.575	0.611	0.135	0.28
Unemployment rate (%)	0.007	−0.079	0.937	0.28

**Fig. 3:**
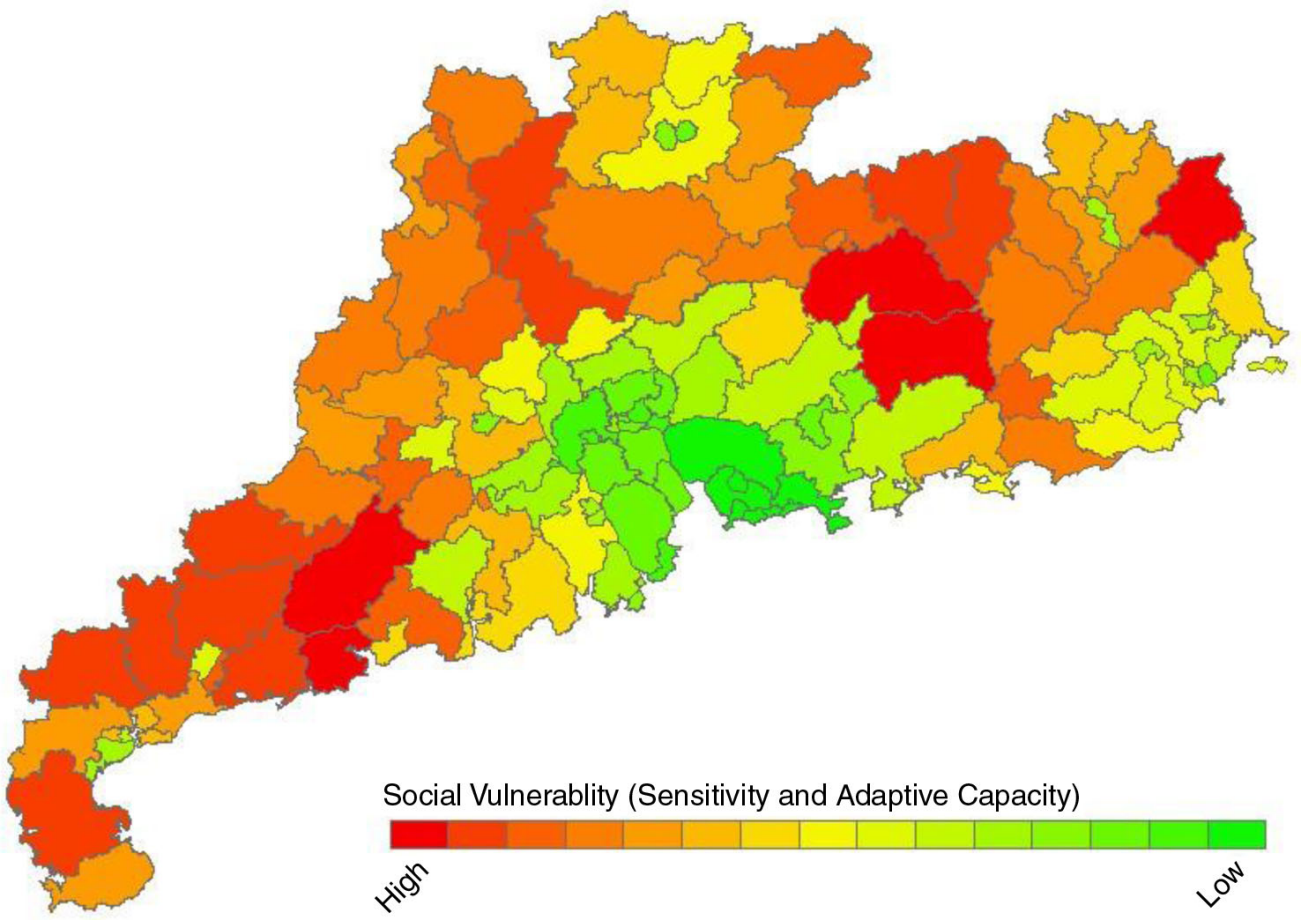
The distribution of social vulnerability to heat waves among 124 counties/districts in Guangdong Province (principal component analysis).

**Fig. 4:**
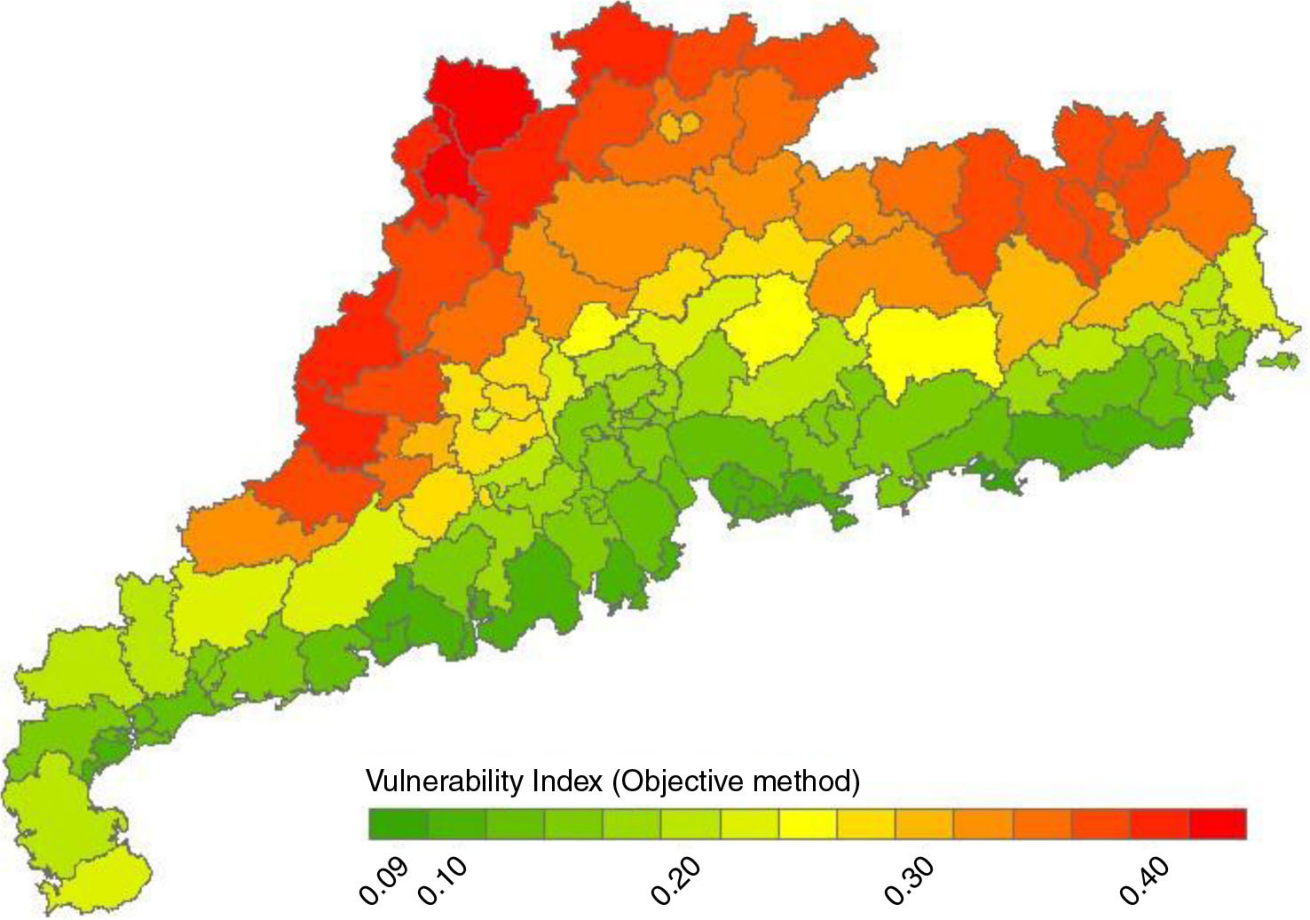
The distribution of vulnerability to heat waves among 124 counties/districts in Guangdong Province (principal component analysis).

Figures 3 and 4 displayed the distribution of SVI and VI to heat waves in Guangdong Province. The Pearl River Delta had lower social vulnerability to heat waves with Yantian district of Shenzhen recording the lowest level. Yangjiang and Heyuan had higher level of social vulnerability to heat waves. Total VI to heat waves of each district/county was assessed based on the SVI and EI. The average VI among the 124 districts/counties was 0.23 with the highest in Lianzhou county of Qingyuan (VI=0.44) and the lowest in Yantian district of Shenzhen and the central district of Shanwei (VI=0.09). The VIs were also gradiently distributed with higher in northern inland regions and lower in southern coastal regions.

### Sensitivity analysis

The ICC for the 124 districts/counties’ vulnerability scores as calculated by the subjective and objective methods was 0.98 with *p*<0.01 (Table 4), indicating that the results were robust to the assessment methods.

**Table 4:** Descriptive statistics of VI calculated by different methods

Assessment method	Mean	SD	Min	Max	P_25_	P_75_
Analytic hierarchy process	0.25	0.11	0.07	0.49	0.16	0.35
Principal component analysis	0.23	0.10	0.08	0.45	0.15	0.32

## Discussion

Research focusing on vulnerability assessment to climate-related hazards has been widely conducted in developed countries across many academic disciplines (31, 32), but few studies have been done to assess the health vulnerability to heat waves in developing countries. To the best of our knowledge, this is the first study to investigate the spatial distribution of health vulnerability to heat waves in China. We observed that the health vulnerability to heat waves was gradiently distributed in Guangdong Province, with higher levels of vulnerability in northern inland regions and lower levels in southern coastal regions. This regional difference may be attributable to several seasons. The southern regions of the Guangdong Province are adjacent to the South China Sea, and as water has a larger heat capacity than land, proximity to the sea could result in the absorption of solar-radiation during hot days, and attenuate the high temperatures (33). In addition, being close to the sea is associated with rain and wind in coastal regions during the summer seasons, which could also reduce the intensity of heat waves. By contrast, most places in the northern inland regions are covered by mountains where heat is not easily diffused. The exposure index in the present study also revealed higher exposure to heat waves in northern regions than that in southern regions. On the contrary, due to the imbalanced economic development, the southern coastal regions had better economic development than the northern inland regions (34, 35), resulting in potentially higher adaptive capacity for communities in the southern coastal regions. For example, people in southern developed regions had a higher average rate of air conditioning ownership than people settled in the northern regions (27) where lack of income was the major reason to stop them buying or using air conditioning during heat waves (36). Although air conditioning has been proved to be one of the most effective measures to reduce health impacts of heat waves (37), it can also increase electricity consumption, discard condensing heat to the air, increase the street temperature, and hence aggravate the heat island effect (38). Therefore, more resources should be allocated to the northern inland regions with higher vulnerability, and a comprehensive adaptation plan should be developed by the local government to improve people's adaptive capacity.

We also observed a large variation of vulnerability in a small area, which is inconsistent to the general distribution of VI in the whole Guangdong Province. For example, in Shaoguan city located in the northern inland region, vulnerability in Wujiang and Zhenjiang districts are significantly lower than that in neighboring districts and cities. This inconsistency is mainly due to the well-developed economy in these two districts, which leads to their low social vulnerability to heat waves (Fig. 3), a finding that illustrates the importance of considering city specific social and economic characteristics when evaluating vulnerability.

Although we did not find similar vulnerability assessment studies to heat waves in China, international studies do offer a point of comparison. The study conducted by Reid et al. in the United States revealed high heat vulnerability in downtown areas and clustering of low vulnerability in outlying areas (17), which was contrary to our findings. Several possible reasons may explain these differences. The United States is a developed country with high level of economic development even in rural areas; people in outlying areas also have high adaptive capacity (39). In addition, Reid et al. mainly focused on the social vulnerability, and did not include exposure indicator in the assessment process. In our study, the vulnerability framework included both social vulnerability and exposure index.

In order to validate the reliability of our findings, a subjective and an objective method were adopted to compare the health vulnerability to heat waves. In the subjective method, the weights of indicators were determined by expert scoring and AHP to avoid unreasonable weight assignment subjectively. In the objective method, a principal component analysis was used to explore the objective inner correlations of different indicators, which may not be easily distinguished by human perception. The results from these two methods were quite similar with ICC=0.98, which implied that experts’ judgment was consistent with the inner correlation of the indicators.

Previous research has suggested the importance of using health data to validate heat wave vulnerability measures and to provide further insights into regional or spatial differences (17, 40). Although we did not assess the relationships between heat waves and health outcomes among all included districts/counties, one of our recent studies revealed that the impact of heat waves on health (mortality) in Nanxiong county (in northern Guangdong Province) was higher than in Guangzhou and Zhuhai city (southern coastal cities) (41). In addition, populations in rural areas of Guangdong Province had lower health risk perception and lower adaptive capacity to heat waves, and had higher prevalence of heatstroke experience than populations in urban areas (37, 42, 43). These results indicated the results of vulnerability assessment in this study is reliable, and can be used for guiding local and regional adaptation policy development.

Several limitations need to be mentioned in this study. First, the construction of the indicators framework and weight determination of each indicator was the critical step within the research process. In the process of indicator selection, some important indicators were excluded because of data unavailability, such as regional population density and prevalence of air conditioning ownership, which may induce bias. It is important to recognize that the introduction of different indicators may have resulted in different outcomes being recorded. Second, only nine experts were invited to weigh each indicator in the AHP and additionally stakeholders from some related fields such as disaster management or clinical practitioners were not included. Again, the inclusion of these into our research process may have resulted in different findings. Despite these potential limits, the results of objective method and health data analyses do provide validity for the general reliability of the overall assessment results.

## Conclusions

The health vulnerability to heat waves had a distinct regional variation in Guangdong Province. Higher vulnerability was observed within the northern inlands compared to the southern coastal regions. This regional variation of vulnerability may be mainly attributed to heat wave exposure and socio-economic variation. In light of the scenarios of the increasing frequency and intensity of heat waves globally in the 21st century, more resources should be promptly allocated to the northern inlands with higher vulnerability. The local government should preferentially develop a comprehensive adaptation strategy to improve people's adaptive capacity on heat waves.
